# Book Reviews

**Published:** 1874-10-15

**Authors:** 


### Book Reviews.

A PRACTICAL TREATISE ON DISEASES OF WOMEN. By T. Gaillord Thomas, M.D., etc. Fourth edition. Philadelphia: Henry C. Lea; Jansen, McClurg & Co., Chicago.

This work is too widely and generally known, through its former editions, to require more than a brief announcement. The present edition has been thoroughly revised, and many chapters entirely re-written, so as to constitute it almost virtually a new work. The author still advocates strongly the lateral method of speculum examination, by the use of Sims' speculum in preference to the cylindrical speculum, introduced in the dorsal position, and all his teachings and instructions are given from this standpoint.

---

A COMPLETE HANDBOOK OF OBSTETRIC SURGERY, OR SHORT RULES OF PRACTICE in every emergency, from the simplest to the most formidable operations connected with the science of obstetricy; with numerous illustrations. By Charles Clay, M.D. From the third London edition. Philadelphia: Lindsay & Blakiston; Jansen, McClurg & Co., Chicago. 8vo., 324 pp. Price $2.25.

The field covered by this little work is sufficiently explained in this somewhat comprehensive title, and the author's name is a full guarantee of its value and reliability.

---

INFANT DIET. By A. Jacobi, M.D. Revised, enlarged, and adapted to popular use, by Mary P. Jacobi, M.D. New York: G. P. Putnam & Sons, Fourth Ave. and Twenty-third Street. Chicago: W. G. Holmes. 120 pp. Price $1.75.

This little volume forms one of Putnam's handy-book series, and contains a plain, common-sense consideration of the subject of infant diet. It is intended and well adapted for the instruction of mothers as well as physicians.

---

OBSERVATIONS ON THE PATHOLOGY AND TREATMENT OF CHOLERA. By John Murray, M.D., of Bengal. New York: G. P. Putnam & Sons, Fourth Ave. and Twenty-third Street. Chicago: W. G. Holmes. 8vo.; flexible cloth covers; 58 pp. Price $1.00.

---

THE TREATMENT OF VENEREAL BUBOES.—Sauszinski (*Centralblatt fur Chirurgie*, No. 6, 1874) has adopted the method of opening buboes by a small perforation, as was advised by Ricord, and later by Zeissel, and has tried it in eighty-two cases of this complication of venereal disease. The bubo is opened with a narrow bistoury, the pus is pressed out through the wound, and it is then dressed with a graduated compress moistened with lead water, over which a small sack filled with sand is laid. The whole dressing is then fastened by means of a Spica bandage, and the patient is confined to his bed for the first few days. At first the compress is renewed twice during the day, but later, when suppuration has diminished, only once, the wound being washed with warm water at each dressing. The sack of sand is used until the edges of the wound become attached to the tissues beneath, when the dressing is changed to charpie and adhesive strips. The advantages claimed for this method of treatment over that by free incision are that the risks of having distinctive ulcerative processes in the wound are much less, and the time needed for its closure is shortened from forty-nine to twenty-eight days.—*Philadelphia Medical Times.*

